# Alzheimer’s Disease: The Past, Present, and Future of a Globally Progressive Disease

**DOI:** 10.7759/cureus.51705

**Published:** 2024-01-05

**Authors:** Bhuvanasai Vejandla, Sarah Savani, Rohith Appalaneni, Rithik S Veeravalli, Sai Sravya Gude

**Affiliations:** 1 Biology, Loyola University Chicago, Chicago, USA; 2 Medicine, Loyola University Chicago Stritch School of Medicine, Chicago, USA; 3 Neurology, Guntur Medical College, Guntur, IND; 4 Medicine, University of Cincinnati College of Medicine, Cincinnati, USA; 5 Pediatrics, State University of New York Downstate Health Sciences University, Brooklyn, USA

**Keywords:** progressive memory loss, new findings, alzheimers dementia, loss of memory, alzheimers disease

## Abstract

Alzheimer’s disease (AD) is a significant 21st-century public health challenge. This article delves into AD's neurodegenerative complexities, highlighting cognitive decline, memory impairment, and societal burdens. Mechanistically, protein misfolding, amyloid-beta (Aβ) pathway abnormalities, and genetic/environmental factors are discussed. The pivotal amyloid hypothesis is dissected, focusing on Aβ aggregation's role in synaptic dysfunction and neurodegeneration. The review showcases promising therapeutic strategies, including anti-amyloid antibodies and β/γ-secretase inhibitors targeting Aβ production. Notably, the FDA-approved Lecanemab signifies a breakthrough, slowing disease progression. Anti-Tau therapies' emergence is highlighted, addressing late-stage intervention. Tau aggregation blockers and anti-Tau antibodies offer potential against intracellular tau pathology. The review underscores collaborative efforts to uncover AD's secrets and pave the way for memory preservation.

## Introduction and background

Alzheimer's disease (AD), an insidious neurodegenerative disorder, stands as one of the most formidable challenges facing public health in the 21st century; it is the leading cause of dementia [1]. With the presence of an aging global population, the prevalence of this devastating condition is steadily rising, it is estimated to affect 106.8 billion people by 2050, making it imperative to delve into its intricate complexities and pursue innovative avenues of research and intervention [2]. Characterized by progressive functional decline, AD robs individuals of their individuality, capabilities, impact on society, and cherished memories and burdens families and societies with an emotional and economic toll of about $321 billion in 2022 [3].

In this comprehensive review article, we embark on an illuminating journey into the world of AD, aiming to present a thorough understanding of its underlying mechanisms, risk factors, and current and future strategies.

Ultimately, the true triumph of AD lies in developing effective interventions that can delay its onset, slow its progression, or, ideally, prevent it altogether [4]. We explore the latest developments in pharmacological, immunological, and lifestyle-based therapeutic approaches that hold promise in mitigating the devastating impact of this condition on affected individuals and their families.

This review will provide a detailed overview of AD and advancement in the research of the diseases within the scientific community. Only through concerted efforts, fueled by empathy and unwavering determination, can we hope to unlock the secrets of AD and pave the way for a brighter future where memory remains a cherished gift for all.

## Review

Disease mechanisms

AD is a protein-conformational disease (PCD) that predominantly results from abnormal processing and polymerization of normally soluble proteins. Because of genetic mutations, environmental conditions, or aging-related protein misfolding, soluble neuronal proteins take on aberrant conformations and cause abnormal neuronal functioning and loss [1]. AD is characterized by the development of extracellular amyloid-ß plaques and neurofibrillary tangles (NFT) in the intracellular environment, neuronal death, and synaptic loss, all of which lead to progressive cognitive impairment [1]. The pathogenesis of AD, a neurodegenerative illness, has been linked to intracellular NFTs composed of hyperphosphorylated tau protein and extracellular aggregates of amyloid ß-plaques in the cortical and limbic regions of the human brain [1]. The documented histopathological features of AD encompass the presence of amyloid ß plaques, which are extracellular aggregates, and NFTs, which are intracellular accumulations of hyperphosphorylated microtubule-associated tau protein. Initially, Aβ plaques emerge in specific brain regions like the basal temporal and orbitofrontal neocortex. As the disease advances, these plaques spread to regions such as the neocortex, hippocampus, amygdala, diencephalon, and basal ganglia. 

Amyloid β pathway

The amyloid hypothesis proposes that AD is caused by the buildup of amyloid-β, leading to synaptic dysfunction and neurodegeneration. Amyloid plaques consist of Aβ peptides, which are derived from the transmembrane amyloid precursor protein (APP). The APP gene is present on chromosome 21 and plays crucial roles in neuron development, synapse formation, repair, and synaptic plasticity in the brain. AD pathogenesis involves proteolytic cleavage of APP-by-APP secretase. There are two processing pathways for APP: the non-amyloidogenic pathway and the amyloidogenic pathway. In the non-amyloidogenic pathway, Alpha (α)-secretase cleaves APP within the amyloid sequence, generating soluble APPα or shorter Aβ species when further cleaved by β-secretase. In contrast, the amyloidogenic pathway involves sequential cleavage by β- and γ-secretase, leading to the formation of soluble Aβ peptides (monomers). These Aβ monomers undergo conformational changes and form stable β-sheet structures, eventually causing pathologic aggregates in the brain, resulting in brain dysfunction and neurodegeneration [1].

The amyloidogenic pathway is regulated by Beta-Site APP Cleaving Enzyme 1 (BACE1), the β-secretase enzyme responsible for cleaving the extracellular region of APP. Mutations in BACE1 (the Swedish mutation and the Italian variant) increase β-secretase activity and have been found in human AD brain extracts. The membrane-bound C-terminal fragment is further cleaved by γ-secretase, releasing Aβ-proteins. Different Aβ peptides are produced depending on the consecutive cleavage of APP by γ-secretase at variable sites, with Aβ42 and Aβ43 having the highest self-aggregating potential. Aβ40, on the other hand, appears to be less prone to aggregation and may even protect against Aβ42 aggregation. These Aβ species, particularly Aβ42 and Aβ40, are abundant in the human brain and play a critical role in AD. Aβ peptides exist in various aggregation states, including monomers, oligomers, and protofibrils, eventually forming fibrils and accumulating in plaques. Among these, soluble oligomeric forms of Aβ are believed to be the main mediators of cytotoxicity in AD and may propagate through an “8prion-like” mechanism [2], where the misfolded Aβ, just like prions could cause deposition of Aβ by recruitment of additional unfolded proteins of a normal protein and causing a cascade and spread through the CNS just as prions do [5].

Type 2 diabetes

Type 2 diabetes has been increasingly recognized as a potential disease pathway for Alzheimer's. Type 2 diabetes mellitus (T2DM) is a complex metabolic disorder characterized by hyperinsulinemia, insulin resistance, impaired glucose metabolism, and eventual pancreatic ß-cell death. In the context of T2D, pancreatic ß cells secrete excess insulin in response to insulin resistance, leading to hyperinsulinemia, which has been associated with an elevated risk of AD. In addition to its role in peripheral metabolism, Insulin acts on insulin receptors that are distributed throughout the central nervous system (CNS), although the precise mechanisms of action of these brain insulin receptors remain unclear. Notably, insulin has well-established effects on the cholinergic system and plays a key role in regulating synapse density to modulate circuit function and synaptic plasticity in the brain. Both hyperinsulinemia and dysfunction of brain insulin receptors have been implicated in the aging process and the pathogenesis of AD. Further research is warranted to elucidate the intricate interplay between insulin signaling in the brain and its potential implications for age-related cognitive decline and neurodegeneration seen in AD [6].

Acetylcholinesterase inhibition 

The initial factor identified in AD is the inhibition of acetylcholinesterase (AChE). AChE comes in two classes: acetylcholinesterase and butyrylcholinesterase, with the former being present in the brain and neuronal synapses. Acetylcholine (ACh) is a crucial neurotransmitter that transmits nerve signals and is vital for learning and memory. The cholinergic hypothesis suggests that in Alzheimer's, there is a decrease in acetylcholine levels due to increased breakdown by AChE into acetate and choline. This reduction in ACh levels affects brain function and contributes to the formation of senile plaques by promoting Aβ aggregation. Therefore, inhibiting AChE is a promising approach for Alzheimer's treatment [7]. 

Tau proteins 

Tau proteins play a significant role in AD pathology, potentially serving as a critical disease mechanism. Under normal conditions, tau proteins bind to tubulin and assist in maintaining the structure of microtubules, essential components for neuronal integrity and function. However, in AD, abnormal phosphorylation of tau proteins leads to a disruption in their interaction with tubulin. This disruption causes a disorganization of microtubules, resulting in alterations to the neuronal structure. Furthermore, these abnormally phosphorylated tau proteins tend to self-aggregate, forming NFTs. The presence of these NFTs is a hallmark of AD and is strongly associated with neuronal damage and cognitive decline. This accumulation and aggregation of tau proteins, along with their impact on microtubule stability, are believed to contribute significantly to the neurodegenerative process seen in AD. Therefore, the dysfunction and aggregation of tau proteins represent a potential disease mechanism underlying the progression of AD [7]. 

Disease risk factors

Numerous studies have suggested the idea that the misfolding, aggregation, and accumulation of protein aggregates play a pivotal role in initiating the pathogenesis of AD [8]. These events are responsible for the subsequent pathological changes that ultimately lead to the clinical manifestations of the disease. A recent study found that healthy lifestyle choices and effective comorbidity management may reduce the risk of dementia, even while the avoidance or prevention of modifiable risk factors may not have a full impact on the future development of the illness [9]. Moreover, aging is the most important risk factor, as an individual with the condition ages, the disease progresses. 

Many vascular diseases, such as hypertension, heart disease (atrial fibrillation, cardiac arrest, or arrhythmia), atherosclerosis, and cerebral amyloid angiopathy, are found to be linked to AD [4]. Cerebrovascular dysfunctions (CVDs) have been observed in the aging and AD brain, indicating a significant variability in the ways these pathologies can impact neurodegeneration and cognitive function. These CVDs play a role in lowering the threshold for developing AD dementia [4]. Cerebrovascular pathologies include cortical infarcts, lacunes, hemorrhages, microbleeds, intracranial small vessel atherosclerosis-arteriosclerosis, and blood-brain barrier (BBB) dysfunction. Extensive CAA and cerebral small vessel disease have been suggested to contribute to neurodegeneration in AD [4]. Additionally, a multitude of microbleeds can significantly contribute to cognitive function decline, while severe white matter lesions are associated with a fourfold increased risk of developing mild cognitive impairment (MCI), which may eventually progress to AD development [4]. Yet, it is essential to note that these alterations are not exclusive indicators of AD; they are also linked to vascular dementia.

Research has indicated that epilepsy may be considered a risk factor for AD [10]. Epilepsy is characterized as a neurological disorder typified by an enduring and spontaneous inclination toward seizure activity, manifesting as either convulsions or non-convulsive episodes, attributable to irregular neural firing and network interactions. Notably, a shared pathological hallmark among epilepsy, sleep disorders, obstructive sleep apnea being most common, and AD is the accumulation of ß-amyloid. Of significant importance, the deposition of Amyloid ß (Aß) plaques and the presence of hyperphosphorylated tau represent the principal pathological features of AD. The accumulation of Amyloid ß results in the disruption of synaptic circuits and neuronal transmission. This disruption not only hampers synaptic plasticity, leading to impairment of learning and memory processes, but also engenders deviant neuronal patterns that can incite heightened network excitability. This augmented network excitability further disrupts the encoding of memories and can trigger modifications in network synchronization. Alterations in the synchronization of neuronal activity coincide with seizure occurrences, wherein an initial desynchronization often precedes the onset of a seizure, eventually culminating in a broader-scale synchronization that intensifies until the seizure's culmination and may even contribute to its cessation of movement and a temporary loss of consciousness [10].

Research findings, particularly the link between Down's syndrome and the triplication of the gene encoding amyloid ß precursor protein (APP), strongly indicate a predisposition towards AD [11]. This genetic association suggests that Down's Syndrome is a significant risk factor for the development of AD. Roughly 40%-80% of individuals with Down syndrome (DS) experience dementia similar to AD by their fifth to sixth decade, which is notably earlier than the typical onset in sporadic AD [11]. The emergence of dementia symptoms in DS aligns with the development of classic brain pathology, such as amyloid plaques, akin to those observed in AD. Both conditions seem to share a genetic connection, evident in the triplication of the gene responsible for amyloid ß (A4) precursor protein (APP) in individuals with DS. In contrast, an additional copy of the APP gene causes familial AD in those without DS. Despite this genetic overlap, the way dementia manifests differs between individuals with DS and those with AD [11]. 

The genes associated with early-onset familial forms of AD are APP, Presenilin 1 (PSEN1), and Presenilin 2 (PSEN2), which have an autosomal dominant inheritance. The sporadic form of AD, which usually occurs after age 60, is most commonly associated with the Apolipoprotein E (APOE) gene [12].

Lifestyle

Diet choices, sleep irregularities, reduced physical activity, smoking, and alcohol consumption can also be considered risk factors for AD. Furthermore, social behaviors such as long periods of isolation and loneliness can cause cognitive decline and are also considered risk factors for AD [13].

Lifestyle choices, including diet, sleep patterns, physical activity, smoking, and alcohol consumption, along with social factors such as prolonged periods of isolation and loneliness, may contribute to the risk of developing AD [13]. 

According to research, a Mediterranean diet (MeDi) lowers the risk of developing AD [13]. MeDi has been suggested for its ability to prevent AD and calls for a low intake of saturated fatty acids like meat and poultry, a low to moderate intake of dairy products from sources like cheese and yogurt, a moderate intake of alcoholic beverages like wine, and a high consumption of unsaturated fatty acids such as fish, vegetables, legumes, fruits, and cereals. Research conducted in Spain, France, North America, and more recently in Australia has shown that stronger adherence to the MeDi is linked to a reduced risk of developing diseases associated with AD risk factors [13]. Additionally, it offers protection against cognitive decline in the elderly population, particularly concerning episodic memory and overall cognitive abilities. This suggests that the MeDi may play a role in lowering the risk of AD. 

However, a recent study of a hybrid of the MeDi and the dietary approaches to stop hypertension (DASH) diet, known as the MIND diet (Mediterranean-DASH Intervention for Neurodegenerative Delay), did not find any significant decrease in the risk of dementia associated with the MIND diet [14].

According to epidemiological studies, circadian rhythm and sleep problems are associated with roughly 44% of AD patients. Though sleep fragmentation can be a normal part of aging, as individuals increase in age, their circadian rhythm and sleep-wake regulation system gradually deteriorate, and their control function weakens, which increases their chance of developing AD [15].

The depletion of cholinergic neurons, a characteristic feature seen in AD patients, has also been documented in individuals who have been exposed to high levels of ethanol consumption. Additionally, there is evidence of hippocampal atrophy, further establishing a connection between intensive alcohol consumption and cognitive impairment that potentially serves as a precipitating factor in the progression toward AD [13].

Current treatment strategies 

Current treatment strategies for AD are primarily targeting managing symptoms and slowing down the progression of the disease. These treatments fall into two main categories: pharmacological and non-pharmacological [16]. 

The first set of pharmacological treatments are acetylcholinesterase inhibitors. The currently approved inhibitors are donepezil, galantamine, and rivastigmine [16]. Their development was rooted in the cholinergic hypothesis, which proposes that the gradual decline of cholinergic innervation in the limbic and neocortical regions of AD plays a crucial role in the deterioration of memory, learning, attention, and other cognitive functions in the brain [17]. The acetylcholinesterase inhibitors increase the availability of acetylcholine at synapses and have been proven clinically useful in delaying the symptoms associated with AD [17]. The primary cholinergic side effects of AChEIs typically affect the digestive system. Healthcare professionals are familiar with these gastrointestinal effects, and they can be reduced by gradually adjusting the dosage and taking the medications with food.

The second set of pharmacological treatments is the N-methyl-D-aspartate (NMDA) receptor antagonist. Excessive activation of NMDA receptors due to glutamate increases excitatory signal transmission, causing excitotoxicity, neural impairment, and heightened excitatory stimulation [18]. Consequently, this results in signaling malfunctions and the gradual degeneration of neurons. Thus, NMDA receptor antagonist memantine has been developed to hinder the consequences of surplus glutamate, in an effort to reinstate the normal transmission of physiological signals in neurotransmission [18].

Non-pharmacological interventions present an alternative, multimodal method slowing the progression of the disease and its symptoms. These interventions center around lifestyle changes more than medication or molecular therapies. For instance, physical activity has demonstrated improvements in terms of physical function, neuropsychiatric symptoms like depression, and the rate of functional decline [19]. Cognitive stimulation programs have shown to be advantageous for sustaining cognitive function and enhancing self-reported quality of life in individuals diagnosed with mild to moderate dementia resulting from AD [19]. However, much of the evidence to support the results of these treatments is still inconclusive, more research is needed to know about the effectiveness of these treatments.

Upcoming and Future Strategies

As discussed earlier, AD is a progressive neurodegenerative condition characterized by a gradual decline in functional and cognitive abilities and the emergence of behavioral problems [20]. However, most current treatments provide only symptomatic relief, and there are no disease-modifying therapies [21]. The recent FDA approval of Lecanemab, the first disease-modifying drug that slows disease progression, presents a promising breakthrough for future therapies.

Disease-modifying therapies

In this section, we explore diverse strategies targeting Alzheimer's pathophysiology at various steps, aiming to slow disease progression and/or even halt the patient's cognitive decline. While we provide an overview of a few avenues, our discussion offers just a glimpse into the extensive landscape of ongoing exploration and research in this field.

 *Anti-Amyloid Therapies*

The Amyloid hypothesis, a leading explanation for Alzheimer's pathophysiology, suggests that abnormal processing of APP by β- and γ- secretases results in the generation of Amyloid-β (Aβ) soluble neurotoxic oligomers, which form fibrils. These fibrils deposit as Aβ plaques in the brain [20]. This Aβ accumulation leads to synaptic dysfunction, neurodegeneration, and symptom development.

Researchers are investigating various drugs targeting distinct stages of Aβ accumulation. One prominent strategy involves anti-amyloid antibodies that remove existing accumulated Aβ. Another avenue is through ß-secretase and γ-secretase inhibitors, which inhibit abnormal amyloid production [22]. 

Drugs that enhance Aβ clearance/anti-amyloid antibodies (Immunotherapy): Anti-amyloid antibodies are monoclonal antibodies designed to clear Aβ. The mechanism behind the action of anti-amyloid antibodies remains uncertain. However, according to the widely accepted hypothesis, these antibodies might traverse the BBB, attach to the amyloid deposits, and aid in their removal through microglia-mediated phagocytosis [23].

Significant research in AD has been focused on anti-amyloid antibodies. The anti-amyloid antibodies have shown significant dose- and time-dependent removal of amyloid plaques in the brain and slowed cognitive decline [22].

Some of the prominent anti-amyloid antibodies include Lecanemab, Aducanumab, and Donanemab. Each of them targets a different stage of Aβ; Aducanumab focuses on Aβ42 oligomers, Lecanemab selectively binds to Aβ protofibrils, and Donanemab targets Aβ plaques.

The two phase-3 clinical trials of Aducanumab (EMERGE and ENGAGE) reported varying outcomes. While the primary endpoint was met in the EMERGE trial, it was not in the ENGAGE trial. Nevertheless, accelerated approval was granted by the FDA, based on the drug's surrogate endpoint of reduction in Aβ plaques in brain PET scans in both trials. However, there's no conclusive evidence linking this biomarker change to improved cognition. Additionally, the FDA mandated a confirmatory trial to prove its efficacy for the drug’s full approval [23].

Lecanemab recently received full FDA approval following its initial accelerated approval. In a phase-3 trial (CLARITY-AD), Lecanemab lowered amyloid burden and reduced cognitive decline over 18 months compared to a placebo in patients with early AD. The CLARITY-AD study is extended beyond 18 months to investigate the long-term safety and efficacy of the drug. Furthermore, ongoing trials (NCT03887455, NCT04468659, NCT05269394) are assessing the safety and efficacy of Lecanemab in various stages of AD, including early stages, preclinical stages, and dominantly inherited AD [24].

Donanemab's recent phase-3 randomized clinical trial (TRAILBLAZER-ALZ2) reported that it significantly slowed disease progression in patients suffering from early symptomatic AD [25].

Though the studies of the anti-amyloid antibodies look promising, there are a few limitations to consider. Firstly, side effects like vasogenic edema and micro-hemorrhages in the brain MRI, which are collectively referred to as Amyloid-Related Imaging Abnormalities (ARIA), were noted [26]. Secondly, it is important to consider the logistical and financial challenges associated with these treatments. For instance, Lecanemab infusions are necessitated biweekly, while Donanemab is required every four weeks. Additionally, the ongoing need to monitor potential side effects through imaging and the possibility of unexpected hospitalizations significantly contribute to the economic burden of these treatments [27].

Inhibition of amyloid production/β-secretase and γ-secretase Inhibitors: Under normal circumstances, the APP protein is cleaved by α-secretase, generating soluble fragments subsequently processed by γ-secretase. However, in Alzheimer's, β-secretase cleaves APP differently, resulting in a larger soluble fragment and a C-terminal C99 peptide. This C99 peptide is then processed by γ-secretase, leading to the accumulation of insoluble Aβ42 and Aβ40 fragments. Inhibition of the β-secretase and γ-secretase could prevent the abnormal amyloid generation [28]. 

γ-Secretase inhibitors: γ-Secretase, responsible for processing the C-terminal C99 peptide into Aβ42 and Aβ40, seemed an attractive target for preventing amyloid fragment buildup. However, its involvement in various pathways, notably involving the NOTCH, has led to substantial toxicity when inhibited. As a result, targeting γ-secretase for Alzheimer's treatment faces challenges due to its role in essential cellular processes, resulting in significant adverse effects [28].

Β-secretase inhibitors/beta-site APP cleaving enzyme (BACE) inhibitors

Unlike γ-secretase, studies involving β-secretase knockout mice suggested minimal involvement of β-secretase in vital cellular pathways. This promising profile positions β-secretase inhibition as a potentially safer therapeutic approach for reducing pathogenic amyloid fragment production and mitigating AD progression. However, serious challenges still exist with respect to the BBB penetration of the BACE inhibitors [28].

The BACE inhibitors Verubecestat and Lanabecestat progressed to clinical trials following success in preclinical studies. In phase I/II trials, Verbecustat displayed a dose-dependent reduction in Aβ42 and Aβ40 oligomers within the cerebrospinal fluid (CSF) without notable adverse effects. However, in phase II/III trials, both exhibited a decline in cognitive function compared to the placebo group. Thus, several trials involving many BACE inhibitors were halted due to their lack of efficacy and safety concerns [23]. Nevertheless, the BACE inhibitors reducing Aβ oligomers within the CSF in phase-I/II trials underscores their potential to slow down the progression of AD [23].

 *Anti-tau Therapies*

Interestingly, tau pathology seems to be correlated more with clinical symptoms, resulting in an increased focus on anti-tau strategies. Various approaches target different steps in tau pathogenesis, with the potential to inhibit aggregation, stabilize microtubules, modulate kinases and phosphatases, and facilitate protein clearance [8].

Tau Aggregation Blockers

Methylene blue has been proposed as a blocker of tau aggregation. It demonstrated significant therapeutic potential in in-vivo trials in transgenic mouse models [9] and generated minimum safety concerns in an exploratory phase-2 trial [8]. This encouraging outcome led to the subsequent investigation of LMTX, a derivative of methylene blue. However, the subsequent two phase-3 trials failed to reveal any substantial cognitive improvement compared to the control group [8].

*Anti-tau Antibodies* 

The anti-tau antibodies attach to the tau proteins outside the cells and stop them from spreading to nearby neurons. This is important because when tau problems start in one neuron, they can spread to others, worsening the disease. The anti-tau antibodies might be able to stop this seeding and help slow down the disease spreading to other regions in the brain [9]. There are currently many trials enrolled in anti-tau antibodies, and the field of tau immunotherapy is occurring at a rapid pace.

Drug-repurposing

Drug repurposing is another promising avenue in the landscape of drug development for AD. It involves the examination of drugs that have already been approved for other medical conditions to determine if they might hold promise in the management of AD. Currently, 39% of ongoing Alzheimer's research focuses on repurposed drugs, spanning categories like hematologic-oncologic, cardiovascular, psychiatric, and anti-diabetic agents [29]. As the safety and pharmacokinetic profiles of these drugs are well-known, Drug repurposing saves significant time and resources involved in drug development. Ultimately, this expedition in the drug development process significantly aids in the quest to find effective treatments for Alzheimer's.

Anti-diabetic Drugs

Emerging evidence suggests that brain insulin resistance plays a significant role in cognitive deficits, opening up the possibility of repurposing anti-diabetic agents in AD [30].

Intranasal insulin in preclinical studies improved cognitive deficits via enhancement of insulin signaling, reduction of Aβ levels, and the alleviation of brain inflammation. A pilot clinical trial demonstrated the stabilization or improvement of cognition in adults with Alzheimer's. However, a more recent randomized clinical trial failed to replicate these cognitive benefits [30].

Metformin, on the other hand, exhibited promising results in enhancing cognition among patients with mild dementia due to AD. However, a population-based study raised concerns as it observed an increased risk of MCI associated with Metformin use. Moreover, Metformin use did not appear to be linked to cognitive test performance [30].

PPAR-γ agonists such as Pioglitazone and Rosiglitazone improved spatial memory in preclinical mouse models. However, a systematic review of clinical studies reported insufficient evidence to support their cognitive benefits [30].

Interestingly, the GLP-1 agonist semaglutide demonstrated promise in preclinical studies and successfully lowered the risk of acquiring dementia by 53% compared to a placebo in people with early AD during a phase-3a trial. Thus, presently, two phase-3 trials, EVOKE (NCT04777396) and EVOKE Plus (NCT04777409), are investigating the safety and efficacy of semaglutide in early AD [31].

## Conclusions

In AD, where memory loss, behavior changes, and functional decline cast a significant shadow, we recognize the urgent need for new and effective treatments. With the global population rapidly aging, the economic burden of this disease is also growing enormously, making it more important than ever to find effective ways to treat it.

Although current treatments can help manage symptoms, they fall short of altering the course of the disease. This is where Lecanemab, an anti-amyloid antibody's full FDA approval, brings hope. In a phase-3 trial, it lowered amyloid burden and reduced cognitive decline in patients with early Alzheimer's compared to placebo over 18 months. Additionally, exciting avenues targeting different steps in the pathogenesis of Alzheimer's in reducing disease progression are under exploration. Drug repurposing on the other hand brings effective treatment options quicker than traditional drug development.

Thus, with the collective stride of patients participating in research and unwavering scientific pursuit by the scientific community, we can safeguard memories and individual functional capacity threatened by AD. In doing so, these fundamental aspects of human experience can endure as precious treasures for everyone in the future.
